# Comparing health status between patients with COPD in primary, secondary and tertiary care

**DOI:** 10.1038/s41533-020-00196-7

**Published:** 2020-09-08

**Authors:** Mieke M. de Klein, Jeannette B. Peters, Alex J. van ’t Hul, Reinier P. Akkermans, Johannes C. in ’t Veen, Jan H. Vercoulen, Erik W. Bischoff, Tjard R. Schermer

**Affiliations:** 1 Department of Primary and Community Care, Radboud Institute for Health Sciences, Radboud University Medical Center, Nijmegen, The Netherlands; 2 Department of Medical Psychology, Radboud Institute for Health Sciences, Radboud University Medical Center, Nijmegen, The Netherlands; 3 Department of Pulmonary Diseases, Radboud Institute for Health Sciences, Radboud University Medical Center, Nijmegen, The Netherlands; 4 IQ Healthcare, Radboud Institute for Health Sciences, Radboud University Medical Center, Nijmegen, The Netherlands; 5 Department of Pulmonary Diseases, Franciscus Gasthuis & Vlietland, Rotterdam, The Netherlands; 6 Science Support Office, Gelre Hospitals, Apeldoorn, The Netherlands

**Keywords:** Outcomes research, Chronic obstructive pulmonary disease

## Abstract

In this study, we compare health status between COPD patients treated in three different care levels in the Netherlands and assess determinants that influence their health status. We applied the Nijmegen Clinical Screening Instrument to measure eight health status subdomains in primary (*n* = 289), secondary (*n* = 184) and tertiary care (*n* = 433) COPD patient cohorts. Proportions of patients with severe problems in ≥3 subdomains are 47% in primary, 71% in secondary and 94% in tertiary care. Corrected for patient characteristics, differences between the care levels are statistically significant for nearly all health status subdomains. The pooled cohort data show female sex, age, FEV_1_ % predicted and BMI to be determinants of one or more subdomains. We conclude that the proportion of COPD patients with severe health status problems is substantial, not just in tertiary care but also in primary and secondary care. Use of detailed health status information may support patient-tailored COPD care.

## Introduction

Chronic obstructive pulmonary disease (COPD) is a preventable and treatable condition that is characterized by airflow limitation^1,2^ and is now recognized to be a complex multi-systemic disease^3^. The goal of COPD assessment is not only to determine the level of airflow obstruction but also the impact of the disease on the patient’s health status (HS). It is well known that there is only a weak correlation between airflow obstruction, symptoms, impairment and quality of life^4,5^. Because of this weak correlation, it is important to focus on the overall of patients with COPD, which consists of four domains: physiological problems, symptoms, impairment in daily functioning, and quality of life^6^.

In several countries around the world (e.g. United Kingdom, United States, Japan, the Netherlands), patients with COPD are treated in primary care by general practitioners (GPs), in secondary care by hospital-based chest physicians and in tertiary care settings (i.e. pulmonary rehabilitation) by multidisciplinary teams led by chest physicians. In the Netherlands, the care for COPD patients is ‘demand-driven’^7^, which means that treatment is tailored to the specific needs of an individual patient. In order to do so, a detailed assessment of HS is a prerequisite.

While the majority (82%) of patients with COPD in the Netherlands are treated in primary care^8^, most of our current understanding of the clinical features and HS in COPD is based on cohorts of patients who have been recruited from secondary and/or tertiary care settings^9^. Consequently, it is largely unknown how HS differs between patients with COPD who are treated in primary care, secondary care and tertiary care, respectively. It is generally assumed that COPD patients in primary care do not experience substantial problems in their HS compared to patients who are treated in secondary or tertiary care. Thus the aims of the present study were to (i) compare HS between patients with COPD treated in primary, secondary and tertiary care and (ii) to assess patient characteristics (including physiological functioning) that potentially influence HS in patients with COPD.

## Results

### Study population

A total of 906 patients were included in the study. Table 1 shows the characteristics of the patients in the primary, secondary and tertiary care cohorts. Compared to the primary and secondary care cohorts, there were more women than men in the tertiary care cohort. Forced expiratory volume in 1 s (FEV_1_) % predicted was lowest in the tertiary care cohort. In all three cohorts, >50% of the patients showed overweight or obesity.

**Table 1:** Patient characteristics of the primary, secondary and tertiary care COPD cohorts in the study.

	Study population			
	Primary care cohort	Secondary care cohort	Tertiary care cohort	*P* value^b^
	(*n* = 289)	(*n* = 184)	(*n* = 433)	
Sex, % male (*n*)	55 (159)	58 (106)	45 (196)	0.005
Age, years	64.5 ± 10.6	63.9 ± 9.5	60.7 ± 8.4	<0.001
BMI, kg/m^2^	26.6 ± 5.5	26.1 ± 5.8	26.0 ± 5.9	0.329
*BMI classes*				0.008
Underweight, % (*n*)	11.4 (33)	21.7 (40)	19.9 (86)	
Normal weight, % (*n*)	33.9 (98)	24.5 (45)	28.0 (121)	
Overweight, % (*n*)	33.6 (97)	26.6 (49)	30.8 (133)	
Obese, % (*n*)	21.1 (61)	27.2 (50)	21.3 (92)	
FEV_1_ % predicted^a^, %	68.5 ± 16.8	63.9 ± 19.1	44.2 ± 19.1	<0.001

Data are presented as percentage (number) or mean ± standard deviation; BMI = body mass index (underweight: <21, normal weight: 21–25, overweight: 25–30, obese: >30).

^a^FEV_1_ % predicted known: primary care cohort *n* = 279 (96.5%), secondary care cohort *n* = 173 (94.0%), tertiary care cohort n = 431 (99.5%).

^b^*p* value from ANOVA test for continuous variables and Chi-square test for categorical variables.

### Differences in HS between the cohorts

Table 2 shows the mean scores on the Nijmegen Clinical Screening Instrument (NCSI) subdomains for the three COPD cohorts. On all subdomains, statistically significant differences were found between the patients who were treated in primary, secondary and tertiary care, respectively. Overall, tertiary care patients reported significantly higher scores on all NCSI subdomains compared to primary and secondary care patients, and secondary care patients reported significantly higher mean scores on all subdomains compared to primary care patients.

**Table 2:** Comparison of health status subdomains as measured by the NCSI between COPD patients treated in primary care, secondary care and tertiary care.

Subdomain^a^	Study population									*p* value^b^
	Primary care cohort (*n* = 289)			Secondary care cohort (*n* = 184)			Tertiary care cohort (*n* = 433)			
	Mean	±SD	95% CI	Mean	±SD	95% CI	Mean	±SD	95% CI	
*Quality of life*										
General Quality of Life	17.2	15.9	15.4–19.1	21.3	16.2	18.9–23.7	28.8	14.9	27.4–30.2	<0.01
Health-related QoL	4.2	1.8	4.0–4.4	5.0	2.0	4.7–5.3	6.1	1.7	6.0–6.3	<0.01
Satisfaction relations	3.0	1.6	2.8–3.2	3.6	2.0	3.3–3.9	3.9	2.0	3.7–4.1	<0.01
*Functional impairment*										
Subjective impairment	9.2	4.7	8.6–9.7	12.5	5.8	11.6–13.3	17.2	5.3	16.7–17.7	<0.01
Behavioural impairment	10.5	11.3	9.2–11.8	18.0	16.7	15.6–20.4	28.8	15.1	27.4–30.3	<0.01
*Symptoms*										
Subjective symptoms	8.0	4.5	7.4–8.5	11.0	4.9	10.3–11.8	13.3	4.0	13.0–13.7	<0.01
Dyspnoea emotions	8.9	3.1	8.6–9.3	11.5	4.6	10.9–12.2	13.6	4.0	13.2–13.9	<0.01
Fatigue	32.6	11.6	31.3–34.0	38.0	10.3	36.4–39.4	42.4	9.5	41.5–43.3	<0.01

Data are expressed as mean, standard deviation (SD) and 95% confidence interval (CI).

^a^The two subdomains from the main domain ‘Physiological functioning’ (i.e., BMI and FEV_1_ % predicted) were included as potential confounders in the analysis.

^b^*p* values are from multilevel linear regression analysis for overall difference between the three cohorts.

After correcting for sex, age, body mass index (BMI) and FEV_1_ % predicted in the mixed model analysis (Table 3), statistically significant differences between the three patient cohorts remained for all NCSI subdomains, except for the subdomain ‘General Quality of Life’ between the primary and secondary care cohorts and the subdomain ‘Satisfaction with relations’ between the primary and secondary cohorts and the secondary and tertiary care cohorts.

**Table 3:** Comparison of the NCSI subdomains, corrected for potential confounders^a^, between COPD patients treated in primary, secondary and tertiary care.

Subdomain^b^	Primary versus secondary care			Primary versus tertiary care			Secondary versus tertiary care		
	Mean score difference	95% CI	*p* value^c^	Mean score difference	95% CI	*p* value^c^	Mean score difference	95% CI	*p* value^c^
*Quality of life*									
General Quality of Life	−3.36	−6.84 to 0.13	0.06	−10.04	−13.28 to −6.8	^c^	−6.68	−10.21 to −3.15	^c^
Health-related QoL	−0.68	−1.10 to −0.26	^c^	−1.77	−2.16 to −1.38	^c^	−1.09	−1.51 to −0.67	^c^
Satisfaction relations	−0.49	−0.92 to −0.07	0.02	−0.84	−1.23 to −0.44	^c^	−0.34	−0.78 to −0.09	0.17
*Functional impairment*									
Subjective impairment	−3.03	−4.24 to −1.81	^c^	−7.47	−8.60 to −6.34	^c^	−4.44	−5.67 to −3.21	^c^
Behavioural impairment	−6.66	−9.85 to −3.47	^c^	−16.62	−19.5 to −13.65	^c^	−9.96	−13.19 to −6.73	^c^
*Symptoms*									
Subjective symptoms	−2.92	−3.93 to −1.92	^c^	−4.81	−5.75 to −3.87	^c^	−1.89	−2.91 to −0.86	^c^
Dyspnoea emotions	−2.34	−3.22 to −1.46	^c^	−4.04	−4.86 to −3.22	^c^	−1.70	−2.59 to −0.81	^c^
Fatigue	−5.24	−7.62 to −2.85	^c^	−9.69	−11.91 to −7.47	^c^	−4.45	−6.87 to −2.04	^c^

Data are expressed as mean score differences and 95% confidence intervals (CIs).

*NCSI* Nijmegen Clinical Screening Instrument, *COPD* chronic obstructive pulmonary disease, *QoL* quality of life, *BMI* body mass index.

^a^Sex, age, BMI and FEV_1_ % predicted.

^b^The two subdomains from the main domain ‘Physiological functioning’ (i.e. BMI and FEV_1_ % predicted) were included as potential confounders in the analysis.

^c^*p* values are from multilevel linear regression analysis, statistical significance defined as *p* < 0.00625 (Bonferroni correction, see ‘Methods’ section ‘Statistical analyses’).

### Severity of HS problems per care level

**Fig. 1:**
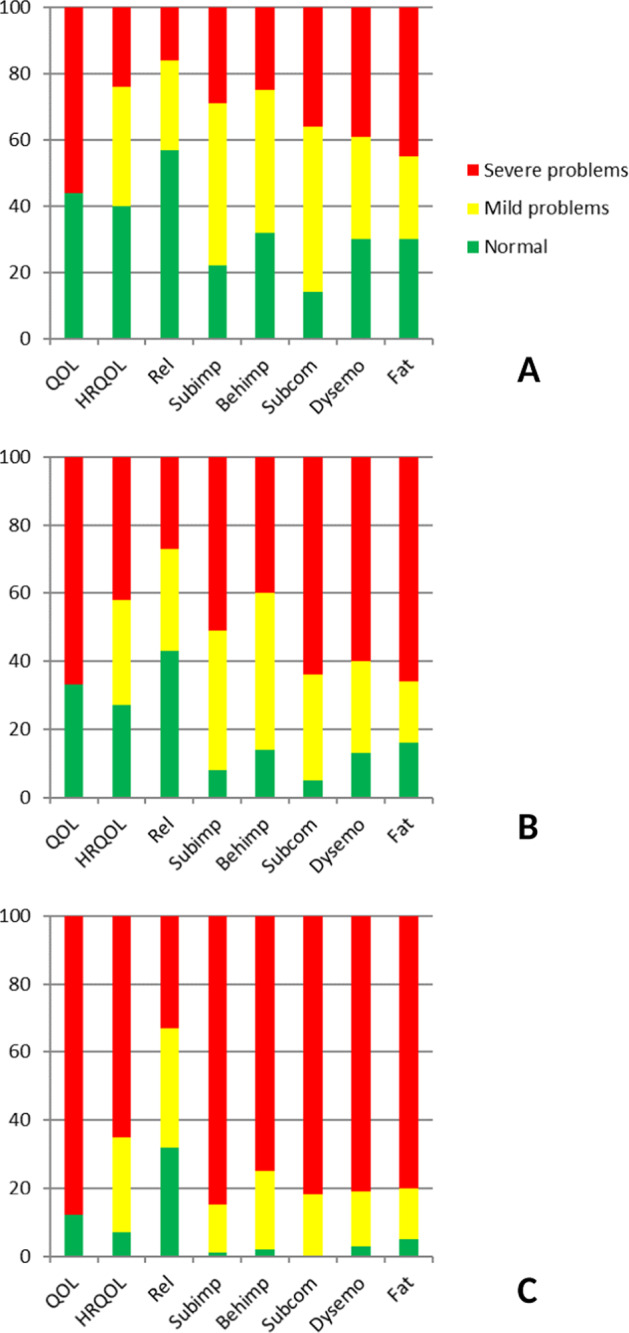
NCSI health status subdomains per care level: distribution of proportions of normal functioning, mild problems and severe problems (%). **a** Primary care COPD cohort; **b** secondary care COPD cohort; **c** tertiary care COPD cohort. QoL quality of life, HRQOL health−related quality of life, Rel relation, Subimp subjective impairment, Behimp behavioural impairment, Subcom subjective symptoms, Dysemo dyspnoea emotions, Fat fatigue, NCSI Nijmegen Clinical Screening Instrument.

Figure 1 shows the distribution of patients with scores in the range of normal functioning, mild problems and severe problems for each subdomain of HS in the three cohorts. Overall, in all HS subdomains the proportion of severe problems was substantially higher in the tertiary care (pulmonary rehabilitation) cohort than in the primary and secondary care cohorts. Increase in the proportions with severe problems when comparing between primary and secondary care cohort was also noted.

**Fig. 2:**
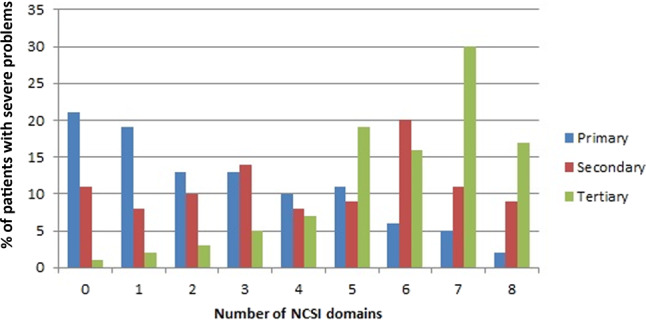
Distribution of percentages of patients with severe problems in the number of subdomains of health status as measured by the NCSI, per care level. NCSI Nijmegen Clinical Screening Instrument.

In the primary care cohort, 21% (60/289) of the patients reported no severe problems in any of the subdomains, whereas 2% reported severe problems in all NCSI subdomains (Fig. 2). In the secondary care cohort, 11% (20/184) reported no severe problems, whereas 9% reported severe problems in all subdomains. For tertiary care, these percentages were 1% (4/433) and 17%, respectively. Severe problems in three or more subdomains of HS were reported by 47% of the patients in primary care, 71% of the patients in secondary care and 94% of the patients in tertiary care (Fig. 2).

### Determinants of HS in the pooled cohort data

The mixed model analysis showed that female patients reported statistically significant more problems in the subdomains ‘Behavioural impairment’ and ‘Subjective symptoms’ compared to male patients (Table 4). Patients who were younger reported statistically significant more problems in the subdomains ‘General Quality of Life’ and ‘Health-related Quality of Life’, ‘Satisfaction with relations’, ‘Dyspnoea emotions’ and ‘Fatigue’ compared to older patients. Patients who were older reported significantly more problems in the subdomain ‘Behavioural impairment’ compared to younger patients. Patients with higher BMI values reported significantly more problems in the subdomains ‘Health-related Quality of Life’, ‘Behavioural impairment’, ‘Dyspnoea emotions’ and ‘Fatigue’ compared to patients with a lower BMI. Finally, patients with lower FEV_1_ % predicted values reported significantly more problems in the subdomains ‘Subjective impairment’, ‘Behavioural impairment’ and ‘Subjective symptoms’ compared to patients with a higher FEV_1_ % predicted value.

**Table 4:** Multivariable analysis of potential determinants of health status in the combined primary, secondary and tertiary care cohorts of patients with COPD.

	Study population											
	Gender: female (ref.)			Age: 1 year younger (ref.)			BMI: 1 kg/m^2^ lower (ref.)			FEV_1_ % predicted: 1% higher (ref.)		
	Effect	95% CI	*p* value^a^	Effect	95% CI	*p* value^a^	Effect	95% CI	*p* value^a^	Effect	95% CI	*p* value^a^
*Quality of life*												
General Quality of Life	−1.42	−3.44 to 0.61	0.17	−0.41	−0.52 to −0.31	<0.01	0.18	−0.00 to 0.36	0.05	0.01	−0.04 to 0.07	0.684
Health-related QoL	−0.16	−0.40 to 0.08	0.196	−0.03	−0.04 to −0.02	<0.01	0.03	0.01 to 0.05	0.003	−0.001	−0.01 to 0.01	0.850
Satisfaction relations	0.11	−0.14 to 0.36	0.378	−0.03	−0.04 to −0.02	<0.01	0.01	−0.01 to 0.03	0.441	0.002	−0.00 to 0.01	0.566
*Functional impairment*												
Subjective impairment	0.04	−0.66 to 0.75	0.908	−0.004	−0.04 to 0.03	0.818	0.05	−0.02 to 0.11	0.142	−0.02	−0.04 to −0.00	0.038
Behavioural impairment	−3.83	−5.68 to −1.98	<0.01	0.35	0.25 to 0.44	<0.01	0.19	0.02 to 0.35	0.024	−0.11	−0.16 to −0.06	<0.01
*Symptoms*												
Subjective symptoms	−0.60	−1.19 to −0.02	0.043	−0.01	−0.04 to 0.02	0.567	0.05	−0.01 to 0.10	0.087	−0.02	−0.03 to −0.00	0.022
Dyspnoea emotions	−0.31	−0.82 to 0.20	0.238	−0.06	−0.09 to −0.03	<0.01	0.05	0.01 to 0.10	0.017	−0.01	−0.03 to 0.00	0.056
Fatigue	−1.04	−2.42 to 0.34	0.140	−0.11	−0.18 to −0.03	0.004	0.18	0.05 to 0.30	0.005	0.01	−0.02 to 0.05	0.455

Data are expressed as effect (i.e. difference in subdomain score), 95% confidence interval and *p* value.

^a^*p* values are from multilevel linear regression analysis.

## Discussion

The main aim of this study was to compare HS between patients with COPD treated in primary, secondary and tertiary care. We also studied several patient characteristics that may be associated with COPD patients’ HS. In the primary as well as in the secondary care cohorts, the proportion of patients with multiple severe HS problems was substantial. Implications of these results are that a substantial part of COPD patients in primary care may require treatment in secondary care (hospital specialist setting) or even tertiary care (pulmonary rehabilitation setting). Or, at least, in these patients a detailed assessment is warranted that in addition to a medical analysis also requires a detailed analysis of HS. Conversely, part of the patients in secondary care may be treated adequately in primary care by their general practitioner. We also found that COPD patients in primary and secondary care showed marked heterogeneity, which means that not all these problems are present in all individuals at any given time point. The marked heterogeneity is found in the type and severity of severe problems in all three main domains of HS: quality of life, functional impairment, and burden of symptoms. Patients in tertiary care showed the most problems in HS, but even in this highly specialized care setting there were also some patients (6%) who showed no or only mild problems in terms of subjective impairment, behavioural impairment and dyspnoea emotions. This small group probably came with limited treatment goals, often physiologically. Overall, our findings indicate that patients with COPD vary from no problems in any to severe problems in all eight HS subdomains. This heterogeneity requires a personalized treatment approach. In addition, the heterogeneity also shows the importance of a detailed assessment of HS in all patients with COPD.

Regarding the first aim of our study (compare HS of COPD patients in three care levels), we traced one previous study by Smid et al. in which COPD-specific HS was compared between the three levels of COPD care^9^. These authors reported that 68% of patients in primary care, 90% in secondary care and 95% in tertiary care were highly symptomatic based on their COPD Assessment Test (CAT) score. Clinical COPD Questionnaire (CCQ) scores (measuring general impact and cough^10^) and St George’s Respiratory Questionnaire for COPD patient scores (measuring HS^11^) also worsened from primary to tertiary care. Patients treated in tertiary care had the worst lung function, more severe symptoms and more impaired HS^9^. Overall, our results that are based on a much more comprehensive assessment of HS using the NCSI are in line with the findings reported by Smid and colleagues.

Regarding our second study aim (i.e. explore potential determinants that may influence HS in COPD), we observed that female patients in our combined cohorts experienced more behavioural impairment and subjective symptoms than male patients. Looking at the existing literature, an explanation for this could be that, in general, female COPD patients experience more problems due to feeling or being responsible for household tasks compared to male patients^12^. On the other hand, COPD seems to have a larger impact on symptoms and physical performance in males than in females^13^. We also observed that younger patients with COPD showed lower quality of life and more burden of dyspnoea emotions than older patients. The literature tells us that younger patients may perceive lower quality of life because of their greater responsibilities related to work, family and/or functional status^14^. Older patients are more likely to change their expectations, may use different reference points to judge their quality of life and may experience less impact of dyspnoea as a result of tolerance of and adaptation to the disease^15^. Previous evidence reported by Berry and colleagues suggests that age modifies how patients with chronic respiratory diseases perceive their impairments and the associated functional limitations, with older patients tending to be more optimistic about their health^14^. In contrast with this, we found that older patients experienced more behavioural impairments than younger patients did. The older patients in our study may have inappropriately attributed their limitations to aging, or possibly (also) to comorbidities.

Finally, we observed that lower FEV_1_ % predicted was associated with functional impairments and subjective symptoms. Psychological factors significantly contribute to disease-specific quality-of-life impairment in COPD and potentially explain the mismatch between objective physiologic impairment and patients’ experience of their disease^16^.

The main strength of our study is the large study population (*n* = 906), combining HS and clinical data from COPD patients who are treated in primary, secondary and tertiary care levels into one study. Another strength is the use of the NCSI method, which provides a detailed and evidence-based approach to study HS in COPD. Assessing a patient’s HS is a prerequisite for personalized COPD management. Other existing instruments usually measure HS of COPD patients in a less comprehensive way^10,11,17,18^. Nonetheless, a limitation of the study is that we did not include other HS instruments (e.g. CAT or CCQ questionnaires) to compare the results of the three COPD cohorts. A final limitation is the lack of (uniform) clinical baseline data on comorbidity and exacerbation rate in the three cohorts. More research is needed to understand which factors also determine a COPD patient’s HS, both in a positive and negative way, in order to achieve better personalized treatment.

In conclusion, our study showed that the proportion of COPD patients with severe problems in HS is substantial. The highest rate of HS problems was seen in patients in tertiary care, but a substantial part of primary and secondary care patients also showed severe HS problems. Knowing that, not all patients seem to be managed at the level of care that would be the most appropriate for them. The pooled cohort data showed female sex, age, severity of airflow obstruction and BMI to be determinants of one or more subdomains of HS. The results of our study imply that detailed assessment of HS is warranted, not only to obtain a better understanding of which care level a COPD patient needs for optimal treatment but also to support healthcare professionals in optimizing and tailoring chronic COPD care.

## Methods

### Study subjects

In this observational cross-sectional study data on COPD, patients’ HS was collected between 2012 and 2017 in three different care settings in the Netherlands: primary care, secondary care, and tertiary care. All data were collected as a part of usual care for the patients involved. We studied the following three cohorts.

#### Primary care cohort

HS data of all 289 patients with COPD receiving care from GPs (i.e. without involvement of a chest physician in the patient’s management) in 8 practices in the general practice network of the Department of Primary and Community Care of the Radboud University Medical Center in Nijmegen were used. In this primary care cohort, patients were included between February 2013 and February 2017.

#### Secondary care cohort

HS data of 184 patients who were referred by their GP to a secondary care chest physician at the Franciscus Gasthuis & Vlietland in the city of Rotterdam were used. The cohort consisted of all consecutive patients with a chest-physician-confirmed diagnosis of COPD referred in 2012, 2013 or 2014, without further selection.

#### Tertiary care cohort

HS data of 433 patients with COPD referred to and enrolled in the Pulmonary rehabilitation programme Dekkerswald of the Radboud University Medical Center, Nijmegen between July 2012 and July 2016 were used. The pulmonary rehabilitation programme is a multidisciplinary intervention consisting of patient-tailored therapies to optimize and maintain physical and psychological condition through physical training and by teaching the patient adequate self-management skills^19^.

Inclusion criteria that applied to all three cohorts were: diagnosis of COPD and age ≥40 years. Patients who were unable to speak or read Dutch and/or had incomplete data were excluded. In case more than one NCSI-based HS assessment had been performed in a particular patient, only the data of the first assessment were used. Data were de-identified at the source before further analysis.

Due to privacy regulations, we could not check whether overlap between the cohorts existed, i.e. whether one or more patients had been included in more than one of the cohorts. This is, however, highly unlikely because (i) the patients in the secondary care cohort were from a very different geographical area (i.e. city of Rotterdam and surroundings in the western part of the country) than the patients in the primary and tertiary care cohorts (city of Nijmegen and surroundings in the eastern part of the country) and (ii) the patients in the primary care cohort had to be managed by their GP only, i.e. without involvement of a secondary or tertiary care chest physician.

Because the aims of our study were explorative in nature, no a priori assumptions with regard to (potential) differences in HS subdomains between the three cohorts were made and a sample size calculation was not applicable.

### Data collection

During routine patient visits, data regarding sex, age, BMI and lung function (i.e. FEV_1_ expressed as percentage of predicted) were systematically collected. The NCSI^6,20,21^ was used to measure patients’ HS. The NCSI is a battery of existing instruments that was empirically composed in such a way that overlap between instruments was avoided and that a wide variety of aspects of HS are measured. Overall, the NCSI measures ten subdomains of HS covering the main domains quality of life (3 subdomains), functional impairment (2 subdomains), symptoms (3 subdomains) and physiological functioning (2 subdomains). Table 5 shows the tests and instruments included in the NCSI. Normative data have been collected in healthy subjects and several cohorts of patients with COPD to identify cut-off scores for normal functioning, mild problems and severe problems^6,21^. For each instrument, the score belonging to 80th percentile of a healthy control population was used as the maximum score of normal functioning, and the score belonging to the 20th percentile of the pulmonary rehabilitation patients was used as the minimum score representing clinically relevant problems^20^. In all subdomains, a higher score indicates more impairment. Patients completed the NCSI before their visit to the general practice, hospital or pulmonary rehabilitation centre, either online at home or using a computer at the healthcare facility. Scoring of the items was automated.

**Table 5:** Subdomains of health status and their definition and included instruments of the Nijmegen Clinical Screening Instrument (NCSI)^1,2^.

Domain	Subdomain	Definition	Instruments/measurement	No. of items	Range
Physiological functioning^a^	Airflow obstruction		Post-bronchodilator FEV_1_ % predicted		
	Body composition		Body mass index		
Quality of life	General Quality of Life	Mood and the satisfaction of a person with his/her life as a whole	BDI Primary Care	7	1–101.6
			Satisfaction with Life Scale	5	
	Health-related Quality of Life	Satisfaction related to physical functioning and the future	Satisfaction physiological functioning	1	2–10
			Satisfaction future	1	
	Satisfaction relations	Satisfaction with the (absent) relationships with spouse and others	Satisfaction spouse Satisfaction social	1 1	2–10
Functional impairment	Subjective impairment	Experienced degree of impairment in general	QoL-RiQ General Activities	4	4–28
	Behavioural impairment	Extent to which a person cannot perform specific and concrete activities as a result of having the disease	SIP Home Management	10	0–135.5
			SIP Ambulation	12	
Symptoms	Subjective symptoms	Patients overall burden of pulmonary symptoms	PARS-D Global Dyspnea Activity	1	2–20
			PARS-D Global Dyspnea Burden	1	
	Dyspnoea emotions	Level of frustration and anxiety a person experiences when dyspnoeic	DEQ Frustration	3	6–24
			DEQ Anxiety	3	
	Fatigue	Level of experienced fatigue	CIS Subjective fatigue	8	8–56

*BDI* Beck’s Depression Inventory, *CIS* Checklist Individual Strength, *DEQ* Dyspnea Emotions Questionnaire, *FEV*_*1*_ forced expiratory volume in 1 s, *PARS-D* Physical Activity Rating Scale-Dyspnea, *QoL-RiQ* Quality of Life for Respiratory Illness Questionnaire, *SIP* Sickness Impact Profile.

^a^Physiological functioning: airflow obstruction and body composition values were used as confounders or determinants of health status in the current study.

### Ethics approval and consent

We applied the Code of Conduct for Medical Research issued by the Dutch Council of the Federation of Medical Scientific Societies^22^ to this research. Because the NCSI and demographic and clinical data were collected as a part of routine patient care and no intervention or course of action was imposed on patients, no ethics approval was required. Patients could refuse the use of their de-identified data for scientific research purposes and have their data removed from the research database.

### Statistical analyses

Descriptive statistics are presented as mean, standard deviation (SD), frequencies and percentages depending on the scale on which the variables are measured. Because of the hierarchical structure of our study (patients nested within general practices and hospitals), we performed multilevel linear regression analysis to test differences between the three cohorts on eight of the ten NCSI subdomains (the two subdomains regarding physiological functioning, i.e. BMI, and FEV_1_ % predicted, were not analysed as such) while controlling for sex, age, BMI and FEV_1_ % predicted. We performed a model with a random intercept and all other variables fixed. A similar model was used to analyse patient characteristics as possible determinants of HS in the combined data of the three cohorts. To avoid increase in type 1 error due to multiple testing when comparing the subdomain scores between the three cohorts, we applied a Bonferroni correction: *p* < 0.00625 (i.e. 0.05/8 subdomains) was considered statistically significant, based on two-sided testing. Analyses were performed using IBM SPSS version 25.0 (SPSS Inc., Chicago, USA).

### Reporting summary

Further information on experimental design is available in the Nature Research Reporting Summary linked to this paper.

## Supplementary information

Reporting Summary

## Data Availability

The data sets generated during and/or analysed during the current study are available from the corresponding author on reasonable request.

## Code availability

SPSS syntax files can be requested by sending an e-mail to the corresponding author.
